# ‘They don’t do nothing’: Exploring marginalized people who use drugs’ experiences with and expectations of private security officers

**DOI:** 10.1177/17488958241249825

**Published:** 2024-05-05

**Authors:** Rachel Geldart, Carolyn Greene, Marta-Marika Urbanik, Katharina Maier

**Affiliations:** University of Alberta, Canada; Athabasca University, Canada; University of Alberta, Canada; The University of Winnipeg, Canada

**Keywords:** Effectiveness, expectations, policing, private security, procedural justice

## Abstract

Private security guards are increasingly supplementing public police in the policing of urban parks. In the context of this expansion, little is known about how people who frequent and depend on access to these spaces, such as street-involved people who use drugs, view and experience private security. Drawing upon 30 interviews and ethnographic observations with unhoused, street-involved people who use drugs in a small Canadian city, findings show that participants held largely neutral views of security. Departing from common perceptions of marginalized people’s disdain towards the social control enacted by law enforcement actors, participants expected and wanted security officers to actively enhance everyone’s safety and well-being. The article concludes with a discussion of the implications of this for security’s legitimacy, and recommendations for improving how private security engage communities.

## Introduction

Scholars have long documented private security’s ever-expanding role in policing (Jones and Newburn, 1999; Rigakos, 2002; Saarikkomäki and Alvesalo-Kuusi, 2020; Shearing and Stenning, 1981). Over the past 50 years, the industry’s growth has been so pronounced that private security officer presence has become commonplace across urban landscapes, prompting frequent interactions between security officers and community members in public (e.g. courts, parks), quasi/mass-private (e.g. malls, car parks) and private spaces (Shearing and Stenning, 1981; Wood, 2020; *see also* Button, 2019). Private security presence has become ubiquitous in public spaces recently, with many governments and other public institutions incorporating private security into their larger public safety strategies (e.g. Button, 2019; Staff, 2021; Van Steden and de Waard, 2013; Wakefield, 2012).

In Canada, security’s progressively dilatant role is perhaps most evident in municipal governments’ growing reliance on private security to supplement public police in the monitoring/policing of urban parks and other green spaces. Formerly the sole domain of the *public* police, many Canadian parks are now patrolled by private security officers who routinely interact with park patrons (Antonio, 2023; Hanrahan, 2022; Petracek, 2022). Yet, encounters with private security officials in these public spaces are not evenly distributed and are often targeted at specific groups. Social and structural inequalities disproportionately subject the most marginalized park-goers (e.g. unhoused persons, People Who Use Drugs (PWUD) to frequent contact with and social control efforts by private security, given these groups often rely on parks as spaces to safely congregate, rest and/or access public bathrooms (Parker, 2019; Rudin, 2018; see also DeVerteuil et al., 2009). This is further exacerbated by the fact that private security’s expansion into many Canadian public spaces – particularly city parks – has been driven by concerns about crime and urban decay. Private park security officers are thus increasingly being tasked with deterring/addressing a variety of social disorder concerns, including disputes, violence, vandalism and open drug use (Maier et al., 2024; *see also* Loader and White, 2018), which renders their encounters with marginalized and unhoused community members commonplace.

Drawing upon interviews with 30 unhoused PWUD in Brandon (Canada), we examine how participants experienced and perceived private security officials working in a public park they spend much of their time in. Working within the parameters of security work orientations (Button, 2007; Kim et al., 2018; Rigakos, 2002), we draw attention to participants’ expectations of how they *desired* security to act vis-a-vis how they *experienced* daily security contact. We argue that participants’ expectations of private security officers’ work fuse elements of multiple and distinctive orientations of security work, and that security officials’ adherence (or lack thereof) to these expectations can shape security–community member dynamics and have implications for safety.

## Private security mentalities and practices

Research suggests private security mentalities and orientations exist on a continuum between the traditional ‘watchman’ and the ‘parapolice’; where security officers sit on this continuum results in considerably divergent approaches to their work activities. For example, in spaces where private security embodies a ‘watchman’ orientation, officers are likely to show disinterest, avoid conflicts and primarily engage in observation and reporting (Button, 2007, 2008). Conversely, the parapolice orientation aligns more closely with public police work and is characterized by officers adopting highly active and committed roles in responding to crime (Button, 2008; Rigakos, 2002).

Recently, scholars have identified two additional private security orientations centred on service or care, which arguably fall somewhere between the ‘watchman’ and ‘parapolice’ approach. In an examination of mall security, Kim et al. (2018) identified a ‘servicemen’ orientation where security officers’ work ‘. . . focused much more on service work rather than security tasks’ (p. 9). Service work included responding to customers’ questions/concerns and providing directions to customers. A separate style of security work emphasizes social care and harm reduction over hardline enforcement. Kammersgaard (2019) unmasked a logic of policing in Danish public spaces frequented by PWUD and marginalized community members that prioritized harm reduction where security officers’ tasks were centred on compassion, including providing a listening ear and/or referrals to social services (*see also* Stuart, 2016). These mid-range orientations change and challenge the traditional scope of private security officers’ work by including and prioritizing tasks such as providing directions to visitors, handling/disposing of bio-hazardous materials and responding to drug overdoses (Kammersgaard, 2021; Maier et al., 2024).

Even in situations where security officers have broadly similar roles and responsibilities, *how* security officers do their work can vary across contexts. For example, Manzo (2004) examined mall security work in Canada, finding security officers’ work orientations were largely shaped by mall milieus. Kim et al. (2018), in a cross-national study of mall security, found that despite holding similar responsibilities, UK security officers generally modelled the parapolice, while their counterparts in South Korea adhered to the servicemen orientation. Context-specific variations in how security approach their work may influence the broader public’s support for private security generally, and, critically, private security’s impacts within the public spaces unhoused community members spend most of their time (DeVerteuil et al., 2009).

Research has long maintained that private security has overwhelmingly served to exclude and displace marginalized populations from quasi-private (e.g. malls, hospitals) and public spaces (Eick, 2003). However, these exclusionary impacts are increasingly being complicated by studies documenting the beneficial impacts of security on PWUD lives (May and Cloke, 2014; Wood, 2020). This suggests that security orientations are an important part of understanding the consequences and effectiveness of private security within specific spatial contexts and across diverse populations. While private security officers are deployed to a broad range of spatial and social contexts, most have limited legal authority. This is particularly important when considering what private security are *expected* to do in public spaces such as urban parks. In Canada, private security officials deployed to and contracted to ‘secure’ public parks have limited formal powers and are often charged with monitoring park facilities and, critically, park patrons’ activities to prevent crime, violence and social disorder. These responsibilities and expectations seemingly clash with officers’ constrained legal authority and raise vital questions about how private security orients itself within and across park settings. Indeed, while park security officer responsibilities may be similar across locations, their approach to their work may vary and thus necessitates additional empirical scrutiny.

Empirical work demonstrates that security work and public perceptions of security are, therefore, context-dependent (Manzo, 2004, 2006) and that local-level research is needed to further identify how context shapes security work and the public’s perceptions of their performance (Moreira et al., 2015; Van Steden, 2023; Wakefield, 2012). In some contexts, private security work that emphasizes social care with vulnerable community members, such as facilitating access to services or checking to see whether medical care is necessary, can lead to marginalized community members viewing security guard presence as immensely beneficial (Kammersgaard, 2021). Yet, such findings are drawn from a limited number of studies exploring how marginalized people who *routinely* encounter, and who are often *targeted* by, park security experience and perceive security officers’ roles and activities within these public spaces (*see also* Herring, 2019; Herring et al., 2020). To date, we know little about marginalized community members’ *expectations* about what security’s role and responsibilities *should* be and, critically, what happens when security fails to meet community members’ expectations within these spatial contexts.

Our findings demonstrate that marginalized community members’ perceptions of park security were generally neutral or ambivalent (*see also* Van Steden and Nalla, 2010). Participants’ narratives highlighted experiences of ‘watchman’-oriented security, which they felt neither benefitted nor directly harmed them. Indeed, our findings demonstrate a considerable misalignment and tension between the security orientation participants experienced and how participants *wanted* park security to behave/work. Participants expected and desired security orientations of care and – to a lesser extent – parapolicing, as they believed these orientations would broadly enhance their safety and well-being on the streets.

## Method

### Setting

This research took place in Brandon, Manitoba (Canada) and is part of a larger, multifaceted Canadian study exploring the views and experiences of PWUD and People Experiencing Homelessness (PEH). Located on Treaty 2 Territory – the *traditional homelands of the Anishanabek, Dakota, Oji-Cree, Cree, Dene and Metis peoples – Brandon is* a small city of 54,268 residents (Statistics Canada, 2023). Despite its size, the city serves as a regional service hub for over 190,000 people living in surrounding communities (Economic Development Brandon (EDC), 2023). Brandon’s population is moderately diverse, with about one in five residents reporting visible minority status (Statistics Canada, 2023).

The researchers conducted fieldwork in the city’s downtown, spending most of their time in Princess Park and areas along Pacific Avenue. Like other cities, Brandon’s downtown has been challenged by economic decline, public drug use and visible homelessness. Downtown Brandon has been the focus of neighbourhood revitalization efforts since at least 2008, and in recent years, the City and Province of Manitoba have dedicated funding to improvement initiatives aimed at attracting ‘prosocial’ people and businesses into the core. Despite these efforts, downtown Brandon continues to face challenges surrounding homelessness and addiction (Kemp, 2022).

In 2021, there were an estimated 139 unhoused persons in Brandon, almost half of who were completely unsheltered (i.e. sleeping outdoors) (City of Brandon (COB), 2021). First Nations, Métis and Inuit persons are disproportionately represented among Brandon’s homeless population. While First Nations, Métis and Inuit represent about 13% of the city’s population, they account for between 74% and 81%^1^ of Brandon’s unhoused population. In Canada, poverty and homelessness are inextricably connected to the Country’s long history of colonial violence and oppression of Indigenous persons (Tetrault, 2023; Truth and Reconciliation Commission of Canada (TRC), 2015).

Many unhoused community members in Brandon spend time downtown where they have access to services (e.g. shelter, soup kitchen, etc.), and specifically, in Princess Park. Throughout the day and into the evening, and unlike most of the streets surrounding it, Princess Park was often bustling with unhoused community members who spent much of their days there or frequently passed through. At times, about 40 people were sitting with friends or enjoying a meal/beverage in the park.^2^ Security guards were observed routinely patrolling Princess Park and adjacent streets. Security officers generally worked in pairs, though at times the researchers observed up to four security guards walking or standing together watching park patrons.

### Data collection and analysis

In the summer of 2022, the authors initiated traditional fieldwork, conducting 30 semi-structured interviews with unhoused PWUD and completing 252 hours of ethnographic observation. The project was approved by the University of Alberta (PRO00127559), University of Winnipeg (HE15863) and Athabasca University’s (23445) Research Ethics Boards. We interviewed 16 men and 14 women. A total of 17 participants self-identified as Indigenous, 10 as white and 3 did not report their race/ethnicity. All but 2 were unhoused at the time of the interview (the remaining 2 were intermittently unhoused). All participants used substances (drugs and/or alcohol). We spent time getting to know people in Brandon, spending many hours hanging out in public spaces bordered by Lorne and Pacific Avenues and 5th and 10th Streets – an area of approximately 21 small city blocks. Once we explained who we were and described the study (including informed consent, confidentiality and anonymity), we invited interested individuals to participate in an interview. Most people we met agreed to be interviewed on the spot or arranged to meet with us later in the day. Participants were compensated with US$30.00 cash for sharing their time and knowledge.

Twenty-nine interviews were audio-recorded and transcribed. One participant declined to be recorded and opted to have the researcher take interview notes instead. Interviews ranged between 9 and 62 minutes, averaging 24 minutes. We deployed a general prompt guide, asking participants a range of questions about their views and experiences of living in Brandon. Prompts encompassed several areas, including feelings of safety, experiences of victimization, harm reduction services, experiences with police and private security officers, healthcare workers, library staff and other community members. We ceased interview recruitment and ethnographic observations when we reached saturation (Charmaz, 2011).

Primarily guided by Braun and Clarke (2006, 2019, 2021), we employed reflexive thematic analysis. Specifically, we engaged in a process of early and ongoing analysis of ethnographic fieldnotes and interview transcripts to identify initial patterns of meaning and organizing concepts (Byrne, 2022). Using a process of open-coding, the researchers collectively and independently identified initial free codes (Braun and Clarke, 2019; Maxwell, 2012), which formed the basis of the researchers’ codebook. In the final stage of analysis, the research team applied the codebook and jointly reviewed and re-reviewed organizational and substantive codes to confirm they represented the data. This article draws from six organizational themes (e.g. park security, policing, victimization) and four substantive themes (e.g. violence in Brandon, street violence, safety strategies).

## Findings

Perhaps surprisingly, participants’ accounts *did not* centre on experiences of security officers harassing, physically harming or displacing them. Instead, participants’ accounts focused on perceptions of and frustrations with private security’s *inaction* in and *disengagement* from the community. Supported by researcher observations, participants described security guards as: (1) avoiding intervention during conflicts, (2) failing to respond to prohibited behaviours (e.g. drinking alcohol, drug use), (3) providing limited/no assistance during medical emergencies (e.g. drug overdoses) and (4) failing to develop relationships with marginalized community members. Perceptions of security *inaction* and *disengagement* did not lead participants to report overly negative *or* positive perceptions of security but rather led to generally neutral assessments of security’s role and impacts. Critically, participants contrasted their current perceptions of security (having little to no impact) against their expectations about what security *should* be doing in and for the community.

### Security’s work in the community

Despite private security’s near-ubiquitous presence, participants described their deployment as generally inconsequential to how and where they spent their time because ‘They really don’t do anything’ (Justina) and ‘They don’t even do their job. Like most of the time they’re on their phone’ (Nico). Although security guards were physically present, participants often claimed their presence was meaningless because they were disinterested in/disengaged from the community and park activities:

They [security guards] don’t do nothing. They just keep to themselves, really. And they don’t really watch ‘cause I know a lot of drinking [is] going on there and they don’t say nothing about that. [. . .] I just see them all sitting together, talking together. Not really looking to see what’s going on or anything. (Angelika)

The other day I walked by and there’s like six of them [security guards] and instead of doing their job, they’re all sitting around talking. They’re all supposed to be around the city, patrolling the city, but no-they’re all up at the park! Just jabbering away, having a sip of water. And it’s like, you guys ain’t doing your job! Like five feet away, there’s a drug deal going down. 10 feet away, there’s people over at the bench drinking. They’re more concerned about their conversation than anything else. (Ricky)

You see them [security guards] around all the time, but they don’t do nothing. Like there’s people drinking in the park, they’ll walk right by. But you know, they’re not supposed to be drinking in the park. They [security guards] don’t care. (Jonathan)

Despite most participants partaking in illicit activities (alcohol and/or drug use) in the park/general downtown area, most criticized private security’s lack of response to illicit activities in the vicinity Courtney, like the participants above, similarly voiced her frustration with security’s inaction, sharing it was not due to security being unaware of these activities: ‘They see everything. They watch everything. They don’t do nothing about it! But they’re there’.

Beyond criticizing park security officials for not actively patrolling the park and responding to illicit behaviours – especially open substance use – Brandon’s most vulnerable community members frequently recounted how security officers routinely avoided or delayed intervening in situations where community members’ health and safety were at risk. For example, they described security officers deliberately avoiding or responding to altercations – which we frequently observed during data collection – in the park:

There was a fight that was happening down here just the other day. Security guards both started walking down here, you know, realized there’s a fight. And I watched them—because I was at the corner—turn around and walk the other way so they didn’t have to deal with it. (Jonathan)

The other day, a fight broke out in the park . . . it took them a while to come and break it up. Like they waited for other people [other park goers] to try break up that fight first. So like, it’s like they tried to avoid confrontation as much as possible. (Shawna)

These accounts are consistent with our observations during fieldwork. We spent significant time in and around Princess Park and witnessed multiple altercations ranging from heated arguments and threats to violent fights. In one instance, two groups in the park were screaming at each other, with one person from each group fighting the other. We observed security *stepping away* from the altercation and choosing not to intervene or attempt to de-escalate the initial argument, even though the argument was slowly escalating. This argument ultimately resulted in a violent fight involving weapons, upon which other park-goers stepped in to try to de-escalate and separate the fighting individuals. It is thus no surprise when we asked Robby whether he thought security officials would intervene in a fight between two marginalized persons, he responded: ‘Not really, no. They just kind of stand there. They don’t do nothing’. Similarly, Ricky explained: ‘They’ll intervene to a point. To a point. They’ll try to, like, avoid it. I’ve seen numerous times where they’ve tried to avoid situations’.

However, participants did provide a caveat to security’s actions, sharing that security officers appear to respond immediately and consistently to any ‘problems’ in the park’s public washrooms. As Anna shared:

The only time they move is when somebody is in the bathroom for longer than 20 minutes, like fuck. So, if you’re constipated then it’s a problem. The homeless people will have their birdbath in there . . . Like you need a few minutes to get cleaned up!

Indeed, when asked what security does when someone is in the bathroom too long, Anna replied: ‘They’ll knock on your door. If you don’t come out right away, they phone the cops’. Arguably, having security check in on people in this context is necessary, especially given the prevalence of drug use (and opioids) in the area and concerns over drug overdoses. However, participants did not believe they could depend on security officers to respond promptly or effectively to medical emergencies. To illustrate, Laura recounted witnessing a woman overdosing in the park and the difficulty she experienced convincing security guards to intervene:

I was like, to security, ‘Come here!’ And they just looked and started talking. I start yelling at them, ‘Get the FUCK over here!’ The security guards were avoiding her [the woman overdosing]. It took them a while and ‘HEY LOOK!’ Then they started running. And I said, ‘Help her!’ They said, ‘We don’t know what to do! We don’t know what to do!’ They were panicking more than anybody.

Laura’s account is troubling because saving someone from a lethal drug overdose – particularly in the context of an increasingly toxic drug supply (Fischer, 2023) – requires a rapid and measured response. While the corporation the security officers worked for indicates employees are trained (basic and site-specific training), participants believed security guards had limited training, especially regarding addressing health emergencies. As Crystal firmly stated: ‘They [park security] don’t know CPR, that’s for fucking sure. They don’t know how to give those Narcan [opioid antagonist] to anybody. Seriously’. We could not confirm whether officers were trained in CPR or Naloxone administration, nor if they were required or chose to carry naloxone. As a result, it is unclear whether the issue was a lack of training, another form of security’s reluctance to intervene or employer-mandated responses. That said, participants’ views are consistent with previous research indicating community members often view private security as poorly trained and ineffective in their work (Moreira et al., 2015).

### Explaining security’s disengagement from the community

For participants, security’s purported disengagement manifested in their failure to ‘do their job’ and/or build more meaningful relationships with community members. These failures contributed to participants feeling security officers did not care about the city or its residents, and especially those who are most marginalized. They consistently reported believing security guards were motivated solely by financial gain and disregarded other aspects of their work and role in Brandon’s ‘tight-knit’ community. Jonathan, who was frustrated at security guards ignoring the open-air drug trafficking, gang posturing and alcohol consumption in the park – curiously, despite engaging in these activities himself – explained, ‘They [security guards] don’t care. They’re there for a paycheck’. Similarly, Tommy emphasized security chooses to avoid intervening to avoid ‘paperwork’, claiming security officers are ‘just there to make their payroll’.

Security’s disengagement was also evidenced in participants’ accounts of the relationships between community members and security officers. Participants were clear, but dismayed that security was not interested in getting to know them and instead ‘ignored them’, only speaking with other security officers. When we asked Adam whether security guards would stop to greet or engage marginalized persons who frequented the park, he replied: ‘Nope. I can guarantee that. They ignore you. They ignore as much as they can’. Similarly, despite spending most of her time at Princess Park, Justina revealed security guards had never approached or spoken to her, nor had she witnessed them assisting anyone. Further illustrating security’s ambivalence towards PWUD, Tommy insisted: ‘They don’t check anybody. They don’t go up to you and be like, “Hey, are you okay?” They just stick by themselves’, adding ‘They’re just standing there all day enjoying the sun and making their payroll’.

Participants perceived security guards’ disengagement was, in part, the result of officer fear, with some reporting security officers were more concerned for their own safety than that of the community. When we asked Matthew why he thought security guards did not address open drug dealing, he responded, ‘Because they’re scared to do something’. Simultaneously critiquing and explaining why security officials would back away from physical altercations, Jose (24 years) told us: ‘It’s their job to like, do stuff, but they probably just—they don’t feel safe either’. Participants cited security officers’ fear as the primary reason they called for police assistance instead of mediating or handling situations themselves. Cassie explains: ‘They’re kind of scared of everything. That’s why [security call police]’. Tommy further revealed: ‘Security doesn’t do anything until police step in’, though he attributed this to the fact that security was ‘. . . not supposed to [intervene]. They’re trained not to touch anybody until police come’. Consistent with Cassie and Tommy’s accounts, we observed security calling the police to the park on multiple occasions during fieldwork.

Finally, despite the overwhelming consensus among participants that security officers were not ‘doing their job’ and did not care about the community, they often described officers treated them relatively positively. Although many participants reported negative experiences with security officers outside of the downtown core, few reported harassment or physical violence. Of the 30 participants interviewed, 2 reported physical altercations with security: (1) security punching a park patron and (2) security putting a park patron in a chokehold. In both instances, community members intervened. Anna describes what happened when her friend was being choked by security: ‘One of his [Anna’s friend] buddies come running there. Had to slug one of the security guards, the one that was choking him’. Dawson recalled that after a security guard punched his family member:

That security guard had to get taken out of there because it wasn’t safe for him. We had that whole park on lock, we were ready, we were ready to go for that security guard because of what he did.

These accounts provide additional nuance. First, they demonstrate the area is not immune to the negative interactions often documented in the literature. Yet, perhaps equally important is that 28 of the 30 participants interviewed did not directly or vicariously experience these negative encounters. When asked how security treat people, Connie explained: ‘They’re not bad. They don’t do anything like that . . . They don’t do much’. These participants even described security positively, though always affirming they fail to ‘do their job’. For example, Marcus shared ‘They’re [security] nice. They’ll try and stop an altercation, but are they able to? No . . . They’re there for fuck all’. When asked if he felt safer with them in the park, he replied ‘I feel safe when I’m around, you know? [Laughing] They [security] don’t do nothing’. Like other participants, Marcus also believed that he could not rely on security to help him if needed, given security’s previous failures to assist marginalized community members. Accordingly, participants relied on each other for assistance: ‘I’d rather go to my friends for security instead of the real security. You know?’ (Monique). Such perceptions and experiences raise questions about the (in)consistency between what private security does and what (diverse) community members expect/desire them to do.

### Security responses, respect and relationships

Underlying participants’ accounts of security officers are their expectations of what participants believed private security *should* be doing. Participants consistently reported a desire for security to take actions that they believed would tangibly and immediately benefit their safety and well-being in the park. They expressed wanting security officers to: (1) intervene in conflicts to de-escalate *instead* of relying on community members and/or police, (2) be adequately trained and a reliable source of safety during emergencies and (3) establish and maintain respectful relationships with park-goers. Indeed, Brandon’s most vulnerable community members shared several ways they believed security officers could/should ‘do their job’ properly, and explicitly connected this to hopes and expectations for more meaningful relationships between them and security officers. For participants, ‘doing the job’ meant responding to community members’ unwanted/prohibited behaviours, intervening and deescalating in altercations/conflicts, responding promptly and effectively to medical emergencies (e.g. drug overdoses) and critically, building rapport with marginalized park-goers.

Our data illuminate several examples of participants’ expectations. When asked what he thought security’s role in the park should be, Terry explained he expects security to intervene when problems arise in the same way that community members do: ‘They should do what we do-Keep shit from happening!’ In response to security failing to do the work necessary to increase community members’ safety, those we interviewed reported they had to rely on themselves and each other to informally police the area. After explaining that security officers ‘just ignore’ community members, Marcus provided additional context: ‘Like I don’t know what they’re [security] there for. I’m like a night security guard for everyone. Because I’m not scared of anyone. And I make sure everyone’s safe, and they’re okay; I protect the females especially’. Similarly, Melissa, when asked if security made the area safe in any way, replied: ‘No, they’re just working for nothing. Because we all keep it safe. There’s always kids around, right? So, we all watch’. Participants further explained that it was community members – not security officers – trying to take the park ‘back’ from some of the disorder occurring there:

The community is actually trying to take it back for the people because – it’s all old folks that come down there and listen to music, so that’s why they put the grandstands up. So, when the addicts are seeing all these old people back in the park, even though they’re addicted, they still have respect. (Anna)

Perceived ineffectiveness and delayed responses to conflict and emergencies were not the only source of security’s delegitimization. Many participants wanted security guards to build relationships with them by getting to know them on a personal level, and central to this was their need for security officers to recognize and respect their lived realities. Despite this desire, and their efforts to foster these connections, participants were adamant that security officers were not interested in building relationships and often ignored/scoffed at their attempts to do so:

I tried to talk to them one day, and they said that they couldn’t talk to the people in the park because that they would get in trouble by their supervisor. And I said, like, ‘Well, how do you expect to get to know the people if you’re not even gonna, like, make an attempt to get to know them? (Brea)

They’re [security is] judgemental. They look at you because you’re drinking, or you get high. Everybody judges based on what they think they know. If they actually took a look into what people go through, why people do what they do. Sit down and actually get to know that person, get to know why they do what they do—maybe then they’d understand. (Roxy)

Notably, rather than wanting security removed from the community for not ‘doing their job’, participants reported a desire for greater security involvement in the park. To be clear, this *did not* mean participants hoped for greater levels of punitive and/or exclusionary social control measures; rather, they wanted security officers to care about and actively help secure their well-being and safety in the park.

Security’s inaction had clear ramifications for how participants perceived them. Specifically, security’s failure to adhere to what the community expected of them diminished their respect for private security. As Terry reveals: ‘If something happens [. . .] and they don’t do nothing about it [. . .] Then they’ll be fucking taunted and shit. [. . .] Everybody always yells at them “Do your job! Do your job!”’ While participants identified security guards’ disengagement and inaction led to a *lack* of respect, they also described how security gained respect by responding to community members’ needs. Tommy explained that when security officials responded quickly to an overdose, ‘Everyone appreciates it. Because they’re looking after our people . . . They get more respect from that’. The benefits of establishing respectful and trusting relationships between security and community members cannot be overstated. Researchers have long documented the importance of this for institutional legitimacy (e.g. police) and, critically, for people’s willingness to cooperate with and obey authorities, especially for those who are marginalized (Grant and Pryce, 2020; Hinds and Murphy, 2007; Madon et al., 2017). We posit that similar dynamics extend to private security officials.

### Park security’s responsibilities in context

To fully contextualize park security work and participants’ views, the government’s and the security firm’s stated role expectations for officers must be considered. At the time of this study, the City of Brandon contracted Paladin Security Services. Although publicly accessible documents outlining security’s role in Princess Park were limited, a procurement document indicated security’s *only* responsibility was regularly patrolling the Park’s washrooms (COB, 2022). If this was park officers’ sole responsibility, participants’ descriptions of park security as ‘watchman’ may have reflected their employer’s contractual obligations. However, the ‘watchman’ orientation appears to conflict with Paladin’s five *basic* employee responsibilities: (1) investigating alarms/emergencies, (2) deescalating crises, (3) supporting emergency services (e.g. police and fire), (4) report writing and (5) providing first aid (Paladin Security, 2023). These outline that, at a minimum, security guards should offer community engagement via de-escalation, emergency response and first aid. Notably, a few months after data collection ended, a new company was contracted to patrol Princess Park. Procurement documents outline that park security are responsible for ‘. . . crime prevention and detection, advising people that are loitering that they must move along, properly disposing of sharps they encounter while on patrol, support and assistance to visitors to the downtown, community support, first aid, [and] police liaison’ (COB, 2022: 7). It is curious to consider whether/how security practices at Princess Park have changed since a new company was contracted.

## Discussion

Perhaps surprisingly – though consistent with findings from other contexts – participants’ views of security officers were neither uniformly positive nor negative and were instead generally neutral (*see* Van Steden and Nalla, 2010). Although most participants did not describe security guards as causing them direct harm (e.g. aggressive/abusive treatment), the absence of overtly negative interactions did not lead to positive views of security. Instead, participants’ neutral or apathetic views resulted from a disconnect between the work security officers did and the work community members expected/desired they *should* do. However, what is important here is that within this spatial context, participants expected an active and engaging security presence in the park. Indeed, participants wanted security officers to simultaneously adopt care/compassion (e.g. get to know community members) *and* enforcement (e.g. intervening in conflict, addressing open drug use) orientations.

Participants blamed park security ‘failures’ on individual security officers’ behaviours rather than on the broader policy and/or structural factors that may have shaped officers’ work (see Button, 2008). It remains unclear whether/to what extent park security officers were officially required to take on responsibilities beyond that of the ‘watchman’, or whether their approach was informed solely by their contractual obligations (washroom patrols). Irrespective of this, participants did report a small number of security interventions (e.g. de-escalation efforts, effectively responding to drug overdoses) arguably orientated in care/compassion and enforcement.

Princess Park’s patrons believed security should be more involved and connected to the community than they were because, like public police, park security officers ‘. . . in their omnipotence and “everywhereness”’ were symbolically viewed as ‘guardians’ responsible for rule enforcement *and* caring, compassionate and respectful relationships with community members (Loader, 1997a: 8). They perceived park security’s ‘watchman’ orientation as idle, ineffective and disengaged from the community, leading many to feel security did not *care* about them or the disorder happening in Princess Park. Despite these negative feelings, participants did not reject security guards’ presence in the area. This finding is consistent with research showing residents of high-crime communities, while justifiably critical of law enforcement, still desire respectful community policing in their neighbourhoods (Pryce and Chenane, 2021).

Participants maintained that park security *could* and *should* take on a more protective function in their work, describing three distinctive expectations about officer’s role and responsibilities. First, participants expected park security to actively respond to obvious rule-breaking (e.g. drug use, fighting). While this may seem counterintuitive, given many participants used drugs in the park, most participants wanted formal rule enforcement. This is likely because when people behave in ways that draw negative attention, it risks public police intervention which threaten their peaceful and safe use of this space. Indeed, those we spent time with perceived a lack of rule enforcement as a security failure and most felt officers were ineffective and undeserving of the community’s respect.

Concerns about security’s ‘watchman’ approach also raised important questions about the potential harms that may result from *under*-policing, including increased violence, distrust, self-policing and/or vigilantism. For example, because security rarely responded to altercations – and when they did, it was at a distance (both physically and interpersonally) – marginalized park patrons were left responsible for intervening or ‘enforcing the rules’. During fieldwork, we witnessed numerous verbal/physical altercations where participants placed themselves at risk of harm as they took on the responsibility for deescalating conflicts.

Participants also expected park security to intervene during all health emergencies promptly and effectively. While first-aid training was a job requirement, officers’ limited interventions led many participants to believe they were inadequately, or not at all, trained to respond to health emergencies. Although we did not witness any serious health emergencies, we did see community members – and *not* security – checking on people they were concerned about (e.g. those appearing to be overdosing/unconscious) and engaged in this ourselves. Indeed, untrained, and highly marginalized community members took on and maintained responsibility for ensuring the health and well-being of other park patrons. Broad perceptions that security did not provide aid in times of need reduced community members’ respect for them and the belief that they were ineffective in their role. Officers’ lack of community engagement and intervention likely contributed to participants’ perceptions. There is opportunity, then, for security officers to earn community members’ respect by identifying and responding to these instrumental needs.

Brandon’s most marginalized community members, as primary park users, also expected security to get to know and build a mutually respectful rapport with them. Establishing positive relationships was so important that some participants reported attempting to initiate these connections themselves. Although it may be surprising that community members desired and believed positive relationships with security were possible, we know that fair and respectful treatment facilitates the development of ‘relational bonds’ (Tyler et al., 2015). Thus, it is plausible security’s generally respectful interactions with participants provided a window of possibility for community members’ getting to know them further and building trust. However, not for lack of effort, participants believed these bonds could not be established because security did not want to or were not permitted to get to know them. Irrespective of security’s reasons for limited community engagement, this had negative ramifications for security–community member relations and eroded participants’ perceptions of security’s work and effectiveness. Ultimately, participants’ experiences and expectations led them to characterize security officers’ observational work as *purposeful inaction.* One reason for this is that security’s ‘watchman’ orientation reinforced ‘. . . negative relational messages of marginalization and neglect’ (Oliveira, 2021: 55; Tyler et al., 2015), leading participants to be sceptical about security officer’s motivations for doing the work, often describing security officers as apathetic about their jobs and the community they were tasked with serving.

Importantly, when security responded to community members’ instrumental needs through respectful and effective interventions, this communicated to park patrons that officers cared for and respected them. This suggests that security officers can earn the respect of community members when their work is aligned with the community’s expectations. Critically, this alignment is the starting point for generating positive, respectful relationships, which can enhance safety for community members and security officers alike (Watson and Angell, 2013; see also Engel et al., 2020, 2022; Mehari et al., 2021; Vickers, 2000; White et al., 2021). This is because “. . . public safety is inexorably connected with the quality of our association with others.”.. and as such, establishing positive relationships between community members and security guards may improve perceptions of security and public safety (Loader, 1997b: 386). Enhancing peoples’ views of security, especially in areas characterized by heightened crime and social disorder, is of particular importance as ‘. . . legitimacy has an important role to play in encouraging desired public behaviors’ and is enhanced by fair and respectful treatment, which ‘. . . promotes rule adherence (Tyler et al., 2015: 100–101). However, in this context, security’s legitimacy depends *on more* than respectful interactions; it also requires meeting community members’ instrumental needs and expectations about what they should be doing in the community (*see* Madon et al., 2017; Murphy, 2013; Murphy and Barkworth, 2014). Indeed, when social control agents respectfully and compassionately interact with marginalized community members – by prioritizing people’s safety over criminalization – marginalized community members are more likely to view law enforcement as a safety resource and critically, access local social/health services (Selfridge et al., 2020; *see also* Greene et al., 2022).

## Conclusion

This research highlights marginalized people’s variegated and nuanced perspectives, experiences and expectations of private security officers in public spaces. Implementation of private security in similar contexts may benefit from emphasizing compassionate care (e.g. relationship building) and respectful rule enforcement over surveillance alone. While private security’s social control function may seem at odds with the caring role of life-saving services, such models have been implemented elsewhere. To further illustrate, recent research from Lethbridge, Alberta (Canada) has found that even when security officers take punitive action to enforce rules in public space (e.g. calling the police and implementing temporary park bans), participants can view and experience security as beneficial to their well-being and thus desire and appreciate their presence in the public spaces they frequent (Maier et al., 2024). This suggests private security can, and likely should, perform both orderly *and* benevolent functions in public spaces. We encourage private security working in similar contexts to consider the benefits of compassionate care approaches for improving people’s perceptions of their work and, more importantly, for increasing safety for all. Arguably, if security officials were to promptly and effectively intervene as participants believed they should, it would likely improve relationships with and foster greater respect from community members.

## Notes

1. The report referenced states 81%. However, the researchers cross-checked this number with the counts and determined it may actually be 74%.

2. While many of the park patrons were street-involved people who use drugs (PWUD), other community members did spend time there (e.g. eating lunch).
